# The Euro Area in Between Crises? Considerations on Fiscal Policies and Rules

**DOI:** 10.1007/s10272-022-1079-9

**Published:** 2022-10-10

**Authors:** Cristina Checherita-Westphal, Sebastian Hauptmeier, Nadine Leiner-Killinger

**Affiliations:** Fiscal Policies Division - Directorate General Economics, European Central Bank, Sonnemannstrasse 20, 60314 Frankfurt a.M., Germany

**Keywords:** E58, H63, H68

## Abstract

Credibility of the revised fiscal rules will be crucial so that vulnerable countries can benefit from confidence effects. National ownership will be key in that respect and can be supported via a stronger role of independent national fiscal institutions.

Fiscal and monetary policy in the euro area acted jointly and forcefully during the COVID-19 pandemic. This allowed economic output to recover to its pre-crisis level faster than many had initially expected given the depth of the blow to the euro area economy. But the support from fiscal policies came at the cost of higher public indebtedness. With monetary policy heading towards normalisation as inflation is at the highest level in decades and the war in Ukraine is on the EU's doorstep, the challenges for fiscal policies at the current juncture are manifold and give rise to some non-trivial trade-offs. They need to continue cushioning the impact from the Russian war against Ukraine and bolster potential output, while not adding to inflationary pressures and debt sustainability risks. The challenges become even more demanding with the deepening of the energy crisis and the continuing deterioration of the economic and inflation outlook. Recent financial market volatility indicates that challenges for fiscal policies are perceived to differ across countries and be related to structural and fiscal vulnerabilities.

This article argues that fiscal policy has an important role to play in shielding the euro area from another crisis. Its tasks are different from those ahead of the euro area sovereign debt crisis, including in the highly indebted countries. But they are neither less nor simpler. To this end, the article stresses the importance of a fiscal framework that is conducive to counter-cyclical fiscal policies, while anchoring expectations that vulnerabilities from high government indebtedness will be gradually yet firmly reduced. To improve the functioning of fiscal rules, it highlights the importance of having the fully independent assessments by strong institutions.

* The views expressed in this paper are those of the authors and do not necessarily reflect those of the European Central Bank. The authors would like to thank Oscar Arce and Mar Delgado Tellez for helpful comments as well as Johannes Simeon Bischl for data support.

**Sebastian Hauptmeier**, European Central Bank, Frankfurt am Main, Germany.

**Nadine Leiner-Killinger**, European Central Bank, Frankfurt am Main, Germany.

## The fiscal response to the pandemic and the war in Ukraine

When the pandemic struck in early 2020, governments were quick to respond in a comprehensive manner at both the national and EU level. This was a new and very different crisis – a health and humanitarian crisis, not one induced by macroeconomic, financial or fiscal imbalances. At the national level, euro area governments implemented emergency and recovery stimulus measures worth about 4% of GDP in 2020 and an additional 0.7% of GDP in 2021. At the EU level, agreement was reached on Next Generation EU, comprising funds worth €750 billion (in 2018 GDP levels) to be spent over 2021-26. Jointly with an accommodating monetary policy, comprehensive support from fiscal policies contributed to an economic recovery that allowed the euro area to surpass its pre-crisis real GDP level in Q4-2021, earlier than many had anticipated. But this came at the expense of higher government indebtedness.

When the Russian war against Ukraine started on 24 February 2022, the euro area economy was thus in a less favourable fiscal position than ahead of the pandemic. In addition, the war brought about an acceleration of energy price increases and pushed inflation to levels not seen in decades, triggering the start of monetary policy normalisation. Once more, fiscal policies were prompt to react to yet another challenging macroeconomic situation. Sizeable fiscal policy measures were enacted to cushion the impact from the war in Ukraine. According to the September 2022 ECB staff projections,[1] euro area discretionary budget support in response to the war in Ukraine is estimated at 1.2% of GDP for 2022. Around 85% of this support is assessed to represent compensatory measures related to increasing energy prices, with the rest relating to defence spending, refugee support and other measures. Based on the government measures approved by early September, one-third of the stimulus is expected to

1 For more details on the measures, based on June 2022 Eurosystem staff projections, see Checherita-Westphal et al. (2022).

Figure 1
**Government debt paths in euro area countries – forecasts and outcomes**

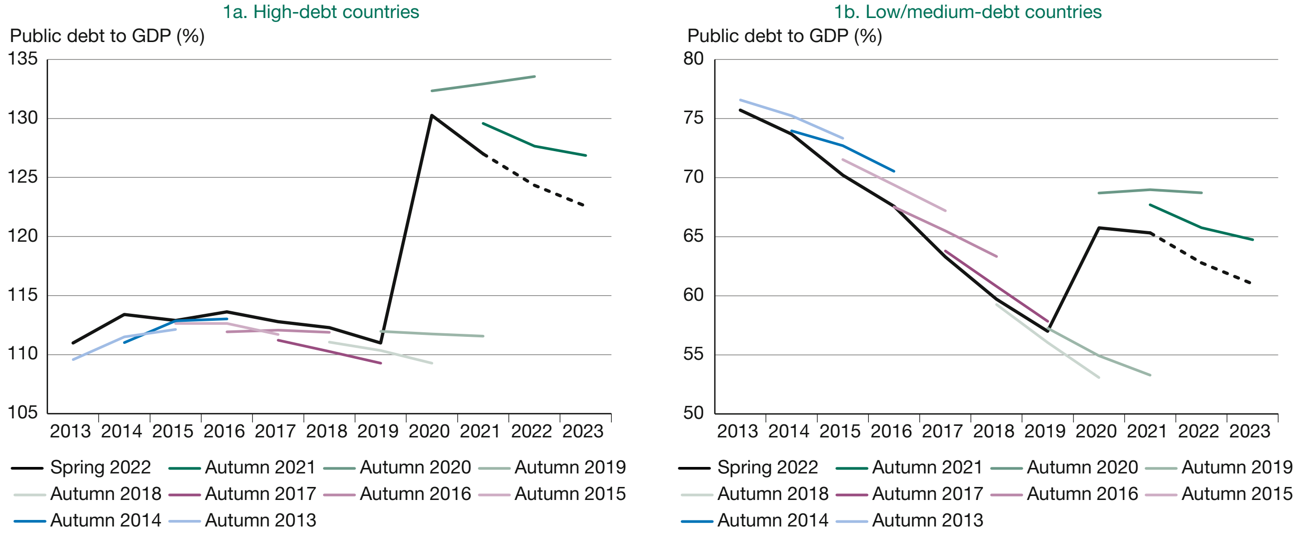

Notes: The government debt outcome (black line) and latest forecast for 2022-23 are taken from the European Commission (EC) Spring 2022 forecast. The coloured lines represent government debt paths as forecast in the EC autumn vintages of the respective year for T; T+2. The (GDP-weighted) aggregate of high-debt euro area countries includes countries with the 2019 government debt-to-GDP ratio above 90%: BE, GR, ES, FR, IT, CY, PT. The (GDP-weighted) low-debt aggregate includes the remaining euro area countries.

Source: Authors' calculations based on European Commission's data and forecast.

continue in 2023, with a large part of the energy support reversing in 2023. Yet, new measures or extensions of the old ones in response to the increasing cost of living are planned or have been already approved by many governments ahead of the Parliamentary debates over their 2023 budgets. The war-related measures currently in the baseline are estimated to have a tangible macro impact. At the euro area aggregate, they are forecast to raise GDP growth by 0.5 percentage points in 2022, with the effect fading out in 2023, and to lower HICP (Harmonised Index of Consumer Prices) inflation by 0.6 percentage points with this impact broadly reversing in 2023.

Most of these measures are estimated to be debt-financed, with some amounts intended to be covered through revenues from the EU Emissions Trading System and relatively limited (albeit more being currently planned) offsetting discretionary measures. On the positive side, better-than-expected cash revenue collections in many countries in the first half of 2022 contribute to lowering the initially forecast deficit impact.

Beyond the measures implemented at the national level, the euro area is estimated to absorb grants from the Next Generation EU programme, especially for a green and digital transition, of 0.6% of GDP in both 2022 and 2023, after about 0.3% of GDP in 2021. These grants do not raise national deficits or public debts immediately, but do raise debt at the EU level. This EU debt will need to be repaid over the longer run.

## Challenges for fiscal policies

The challenges for fiscal policy that arise from a weak macroeconomic outlook, high inflation and elevated debt levels obviously differ across countries. Recent financial market volatility indicates that challenges for fiscal policies are perceived to relate to vulnerabilities from very high government debt, in particular. Countries with elevated government debt-to-GDP ratios will need to focus on improving fiscal sustainability, which will require both sizeable, though gradual, fiscal adjustments as well as solid and sustained growth over the medium term.

This is easier said than done. Looking back at the past decade, Figure 1a shows that highly indebted countries (defined as having recorded a pre-pandemic debt-to-GDP ratio above 90%), on average, struggled to firmly lower public debt ratios even in more normal times following the sovereign debt crisis. As shown in the figure, their debt path was broadly flat over 2013-19, while countries with low and medium levels of indebtedness managed to bring debt ratios on a sustained downward trajectory. In fact, government debt paths in the higher indebted countries, on average, turned out to be consistently above forecasts (while the opposite holds, on aggregate, for the

Figure 2
**Structural adjustment, interest payments and public investment in selected euro area countries**

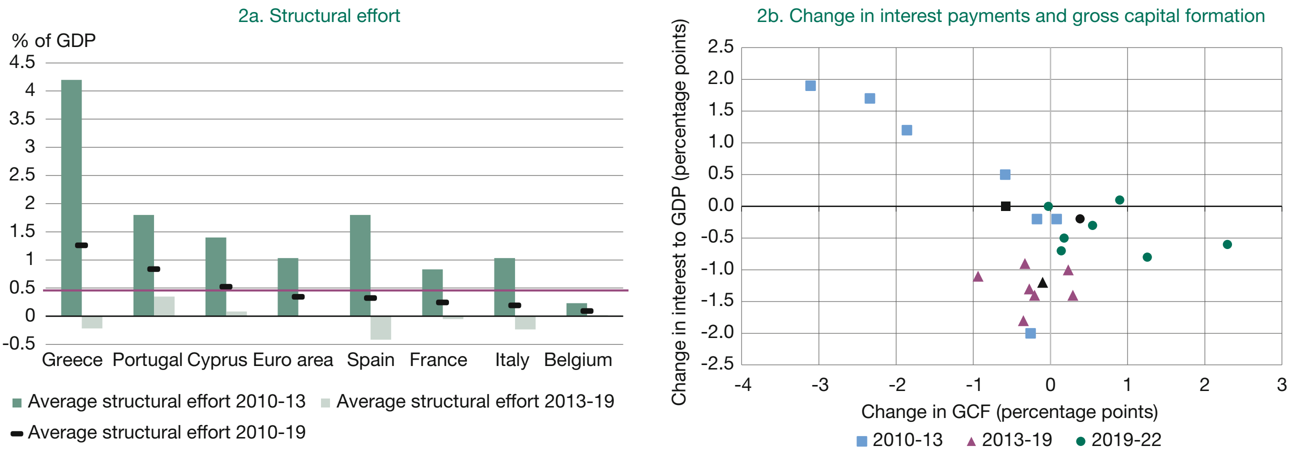

Notes: The figures show countries with public debt-to-GDP ratios above 90% in 2021. In Figure 2a, the horizontal line refers to the Stability and Growth Pact's benchmark requirement of structural effort, i.e. 0.5% of potential output. In Figure 2b, the black points refer to the euro area aggregate, while the other points refer to the higher-debt countries, as identified in Figure 2a (all points denote average values over the periods shown in the legend). GCF stands for public sector's gross capital formation.

Source: Authors' calculations based on European Commission's data and forecast.

remainder euro area countries). This limited debt reduction also reflects rather small or in part negative structural efforts during this period (see Figure 2a), which followed the sizeable and painful adjustments during 2010-13. Once the pandemic hit in 2020, indebtedness increased significantly in the high-debt countries (and far more than in the rest of the euro area).

By contrast, in 2021, once the euro area learned to live with the pandemic and forged itself out of the crisis, debt ratio developments were relatively more favourable in the more indebted countries, due to strong denominator effects related to the exceptional rebound in nominal growth. As shown in Figure 1, debt ratio outcomes during the immediate euro area's post-pandemic economic rebound generally surprised on the positive side, turning out lower than forecast.

The important role of economic growth in supporting debt reductions is a reminder that episodes of fiscal consolidation should keep the economy's growth potential intact. Maintaining or even expanding public and private investment, accompanied by growth-enhancing structural reforms, is vital for safeguarding potential output and thus debt sustainability. This is a major lesson from the sovereign debt crisis, which during 2010-13 saw reductions in gross capital formation as interest spending rose on increasing debt ratios (see Figure 2b). However, despite the more normal economic times over 2013-19, the persistent decline in interest expenditure was not used to raise gross capital formation in many more highly indebted countries. In fact, by 2019, several governments' public investment-to-GDP ratios were below their levels in 2013.

According to the European Commission's 2022 spring economic forecast, the trend decline in government investment in more highly indebted countries appears to have come to a halt. For 2022, in several of these countries, the level of gross capital formation in percent of GDP is expected to be higher than in 2019, although the decline in interest spending is forecast to end. This favourable development is also a reflection of the impact of Next Generation EU, the main instrument of which, the Recovery and Resilience Facility (RRF), is benefiting some higher indebted countries. Figure 3 shows that the per capita allocation of RRF grants is tilted towards countries, which, according to the Commission's most recent assessment, are subject to relatively larger risks to debt sustainability (European Commission, 2022).

These positive developments are subject to two major risks though. First, the share of governments' budgets that will need to be allocated to interest spending is likely higher than forecast in the Commission's 2022 spring economic forecast as monetary policy continues its path towards normalisation in light of historically high inflation. This may put downward pressure on planned government investment that is unrelated to the RRF. Second, the absorption of grants under the RRF may turn out to be less than initially expected. So far, there is mainly anecdotal evidence that some governments are having difficulties absorbing the EU funds that they have been allocated. The

Figure 3
**Recovery and Resilience Facility allocation per capita and fiscal sustainability, euro area countries**

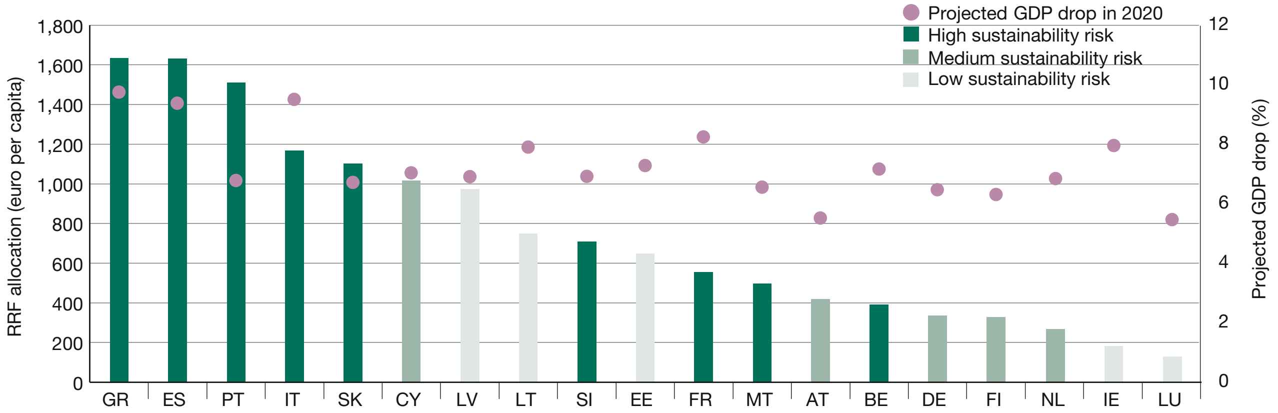

Notes: Medium-term fiscal sustainability risks according to the European Commission's 2021 Fiscal Sustainability Report. Projected GDP drop denotes the real GDP growth rate for 2020 (inverted scale) according to the European Commission Spring 2020 forecast.

Source: Authors' calculations based on European Commission's data and forecast.

main hurdles appear to be related to constraints in administrative capacity, supply bottlenecks in the current post-pandemic recovery and energy crisis, as well as the need to redraft public procurement due to higher than initially planned costs of energy. Moreover, in light of rising interest rates, loans are also becoming more expensive under the RRF.[2] While the relative cost compared to nationally-issued debt may still be lower for some countries, this is likely to reduce countries' already contained incentives to make use of them. This would further deteriorate the outlook for RRF-related investment.

A particular challenge for fiscal policies in the euro area at the current juncture relates to the high inflation environment. Different from the fiscal response during 2008-13, the impact of high inflation especially on households with low incomes requires governments to provide them with support beyond the usual automatic stabilisers that start working as the economy slows and unemployment rises. Where additional public support is required to cushion the impact from the war in Ukraine and from high inflation, financial resources should be used efficiently. In particular, in order to avoid contributing to aggregate demand and high inflation, fiscal measures need to be temporary and increasingly targeted at compensating the most vulnerable households from excessive increases in the cost of living.[3] Generally, increasing the resilience to future shocks in this difficult economic and social environment may require governments to resort to policies that have already proved difficult in the past. Beyond this, more innovation eased by structural reforms, including that which reduces bureaucracy, will be elementary for raising potential output growth and thus fiscal sustainability. The current challenges related to, inter alia, climate change should provide a natural boost for more innovation and investment, including from the private sector.

## The role of the fiscal framework

A well-functioning fiscal framework is an important ingredient for sound fiscal policies. It appears particularly important in times of high uncertainty and in the presence of vulnerabilities to shocks. Such a framework can support fiscal policies in two important respects. First, if credible, it can anchor expectations that high government debt will be brought down gradually yet firmly. Second and related, it can ensure that fiscal policies maintain sufficient counter-cyclical properties. There is broad-based agreement that the functioning of the EU's Stability and Growth Pact

2 Next Generation EU (NGEU) allows the EU to issue a significant volume of debt at the European level. Specifically, the issuance of new NGEU debt takes place between mid-2021 and 2026 in the form of bonds of up to €150 billion. The Commission takes a role in the capital markets as a major provider of safe (AAA-rated) assets denominated in euro. For more details, see Bankowski et al. (2022).

3 This includes a review of whether some more broad-based measures enacted to tackle the crises are still warranted and/or adequate. For the time being, only about 10% of the total energy measures included in the September 2022 ECB staff projection baseline (in terms of their budget impact at the euro area aggregate) are assessed to be specifically targeted to low-income households (means-tested). Discussions are currently on-going in many countries to make this support better targeted to the most vulnerable households.

(SGP) can be improved. In particular, it could better ensure that economic good times are used to build buffers, thereby reducing vulnerabilities from high government debt and creating scope for more counter-cyclicality.

The EU's economic governance review,[4] which the Commission launched in February 2020, has resulted in a broad-based discussion on avenues for reforms. An important element of this debate is how the effectiveness of the fiscal framework can be improved. The reform of the EU's fiscal framework in 2011 foresaw a strengthening of the European Commission in enforcing the fiscal rules via the principle of reverse qualified majority voting in the Council in case member countries disagreed with the recommendations from the Commission. However, in the end, this reform has made it more difficult for the Commission to put forward decisions under the SGP that are unpopular in the Council and thus likely to be rejected. Therefore, a promising avenue for improving the implementation of an economically meaningful framework of fiscal rules that has sufficient ownership across the EU member states is more recourse to an independent assessment by the national fiscal Councils and at the EU level through the European Fiscal Board.[5]

There is evidence that a stronger role of Independent Fiscal Institutions (IFIs) matters (Căpraru et al., 2022; Beetsma et al., 2019). For example, their role in scrutinising the macroeconomic forecasts underlying countries' stability and convergence programmes, which came with the so-called 2-pack regulations, has raised these projections' reliability. In this respect, IFIs tasks could be extended to, inter alia, also include the cost of budgetary measures. Generally, any reduction in the density of common fiscal surveillance at the EU level that is accompanied by more accommodation for country-specific eventualities would benefit from a strengthened role of IFIs. In this context, Barnes (2022) argues in favour of increasing the scope of involvement of national IFIs in the implementation of the EU fiscal governance framework, advocating also that the EU level should be legally required to take into account the assessment of national IFIs. A more effective involvement of national IFIs will take time, however, given the existing large heterogeneity of the scope and capabilities of these institutions.

## Conclusion

Fiscal policymakers in the euro area are facing extraordinary challenges in a low-growth, high-inflation environment. Significant investment needs arise from, inter alia, the green and digital transitions. The war in Ukraine adds to these challenges in the short and longer term. At the same time, several countries are fiscally constrained due to debt ratios standing at record highs following the COVID-19 pandemic. With the ongoing monetary policy normalisation putting upward pressure on interest rates, anchoring market expectations regarding public debt sustainability has become an additional policy objective in high-debt countries. In this context, this article argues that a reformed SGP framework that effectively supports counter-cyclical fiscal policies, notably the building of buffers in good times, will be conducive to macroeconomic stability in the EMU. Credibility of the revised fiscal rules will be crucial so that vulnerable countries can benefit from confidence effects. National ownership will be key in that respect and can be supported via a stronger role of independent national fiscal institutions.

4 See European Commission (2021). In its Communication, the Commission reiterated that the European fiscal framework should ensure debt sustainability while promoting sustainable growth through investment and reforms. To that end, fiscal surveillance should pay more attention to the medium term, also drawing on the insights from the governance and operation of the RRF. The Commission also stressed that the rules should become simpler with stronger national ownership and better enforcement as key objectives.

5 Also the International Monetary Fund, in a recent contribution to the debate, emphasised the need for a reinforcement of the link between European fiscal rules and national budget implementation, notably through enhanced mandates of national independent insitutions. See Arnold et al. (2022).
